# The Correlation of Ten Immune Checkpoint Gene Expressions and Their Association with Gastric Cancer Development

**DOI:** 10.3390/ijms232213846

**Published:** 2022-11-10

**Authors:** Danzan Mansorunov, Natalya Apanovich, Fatimat Kipkeeva, Maxim Nikulin, Olga Malikhova, Ivan Stilidi, Alexander Karpukhin

**Affiliations:** 1 Research Centre for Medical Genetics, 1 Moskvorechye St., 115522 Moscow, Russia; 2 Blokhin National Medical Research Center of Oncology of the Ministry of Health of Russia, 24 Kashirskoe Shosse, 115478 Moscow, Russia

**Keywords:** immune checkpoints, expression, correlation, gastric cancer

## Abstract

In the immunotherapy based on immune checkpoint inhibition (IC), additional ICs are being studied to increase its effectiveness. An almost unstudied feature is the possible co-expression of ICs, which can determine the therapeutic efficacy of their inhibition. For the selection of promising ICs, information on the association of their expression with cancer development may be essential. We have obtained data on the expression correlation of *ADAM17*, *PVR*, *TDO2*, *CD274*, *CD276*, *CEACAM1*, *IDO1*, *LGALS3*, *LGALS9*, and *HHLA2* genes in gastric cancer (GC). All but one, *TDO2*, have other IC genes with co-expression at some stage. At the metastatic stage, the expression of the *IDO1* does not correlate with any other gene. The correlations are positive, but the expressions of the *CD276* and *CEACAM1* genes are negatively correlated. The expression of *TDO2* and *LGALS3* is associated with GC metastasis. The expression of *TDO2* four-fold higher in metastatic tumors than in non-metastatic tumors, but *LGALS3* was two-fold lower. The differentiation is associated with *IDO1*. The revealed features of *TDO2*, with a significant increase in expression at the metastatic stage and the absence of other IC genes with correlated expression indicates that the prospect of inhibiting *TDO2* in metastatic GC. IDO1 may be considered for inhibition in low-differentiated tumors.

## 1. Introduction

Inhibition of immune checkpoints (ICs) is considered as one of the most promising methods of cancer immunotherapy. Such inhibition leads to antitumor activation of the immune system due to the elimination of the IC blocking effect. At present, inhibition of PD-L1, PD-1 and CTLA-4 is mainly used in medical practice. Despite the high efficiency of the treatment achieved in some cases, the proportion of patients responding to such immunotherapy is not yet large. In this regard, other ICs are being explored, and understanding the criteria for identifying the most promising of them can contribute to a faster advancement of research in this direction. Our analysis of published data, both in terms of the effect of IC inhibition and the relationship of their expression with the clinical characteristics of tumors, led to a conclusion that there is a relationship between the properties of ICs as participants in cancer development and the properties that determine the activity of the immune system during their inhibition [1,2].

The presence of ICs, which may be expressed simultaneously with inhibited ICs, can lead to a decrease in the effectiveness of the immunotherapy due to their blocking of the immune system. This circumstance may be one of the reasons for the reduced proportion of patients responding to IC inhibition. This issue is practically unexplored.

Based on the circumstances described, we performed an appropriate examination of gastric cancer (GC) samples. GC is one of the most common cancers, ranking 5th for incidence and 4th for mortality globally [3]. It is difficult to cure and is characterized by a low survival rate, so the development of an effective therapy for this cancer is especially urgent [4].

The expression of 10 IC genes—*ADAM17*, *PVR*, *TDO2*, *CD274*, *CD276*, *CEACAM1*, *IDO1*, *LGALS3*, *LGALS9*, and *HHLA2*—was studied. The selection of these genes was based on the available data on the results of inhibition of the ICs encoded by them and their association with some clinical characteristics, mainly survival, of cancer patients. At the same time, the association of these genes with metastasis and other features of the GC development has not been sufficiently studied [1]. There is practically no data on their co-expression. The expression of these genes was investigated in the early stages of GC development and during metastasis. At these two stages of GC development, the expression correlations of the above genes were studied.

## 2. Results

### 2.1. The Association of IC Gene Expression with the Development of Metastases

**Figure 1:**
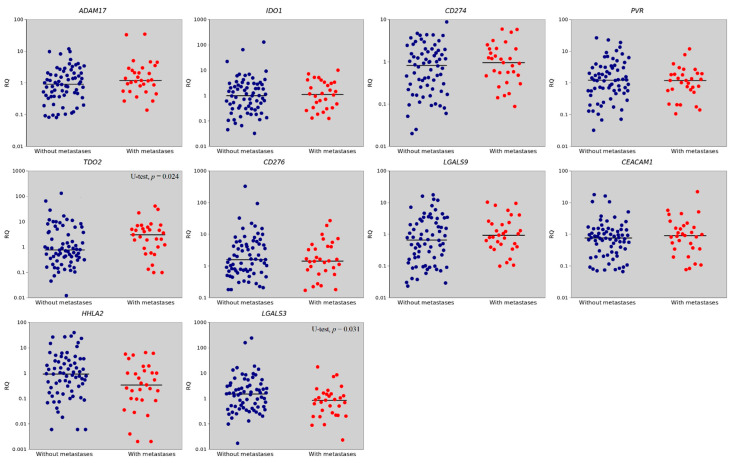
The relative gene expression (RQ) in groups with metastases (red dots) and without metastases (blue dots). Gene expression values are presented on a logarithmic scale. The line marks the median.

The expression level of 10 IC genes was determined in 101 paired stomach tissue samples (tumor/normal). Among the genes studied, expression levels in non-metastatic tumors were slightly higher than in normal tissues for *PVR*, *CD276* and *LGALS3* genes. In metastatic tumors, *TDO2* expression was higher (*p* = 0.024) and *LGALS3* expression was lower (*p* = 0.031) relative to tumors without metastases. The expression of the remaining genes did not exhibit statistically significant changes (Table 1, Figure 1).

To characterize the relationship of the expression levels of these genes with metastasis, ROC analysis was used (Table 2). The analysis revealed that the expression of the *TDO2* and *LGALS3* genes had a statistically significant relationship with GC metastasis. The significance of the differences was retained when applying the Benjamini–Hochberg procedure for multiple comparisons (FDR). That is, an increase in the expression level of the *TDO2* gene was an unfavorable prognosis for the development of metastases. For the *LGALS3* gene, an unfavorable prognosis was associated with a decrease in its expression level.

Differences in the median of expression level and ROC analysis showed a relationship between *TDO2* and *LGALS3* genes expression and GC metastasis. In order to evaluate this relationship, we obtained odds ratio (OR) and relative risk (RR) values and determined the relationship between gene expression levels and metastases using Fisher’s exact test. ROC analysis revealed the cut-off values for expression levels in non-metastatic and metastatic GC that exhibited the best sensitivity and specificity (Table 2). For each gene, the frequency of expression was determined to be higher/lower the cut-off value in GC with and without metastases (Table 3). According to the results of the Fisher’s exact test and 95% CI for OR and RR, there was a significant association of the *TDO2*, *LGALS3* and *LGALS9* expression level with the metastasis. The OR for these genes ranged from 4.2 to 6.6, and the RR ranged from 3.0 to 3.5, with a minimum value of 95% CI greater than 1. The highest OR and RR values were found for the *TDO2* gene.

### 2.2. The Correlations of Gene Expressions

**Figure 2:**
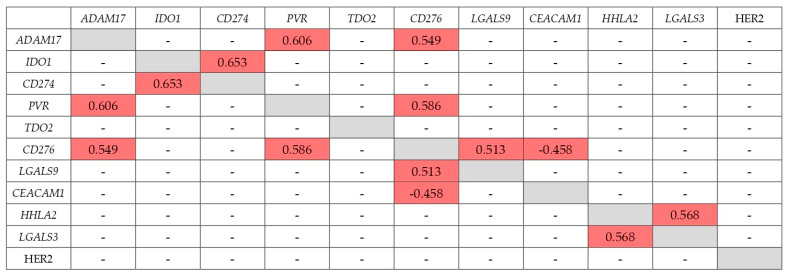
Correlation of expression at stage I + II (early GC). 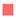 significant correlation; 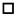 there is no sig-nificant correlation.

**Figure 3:**
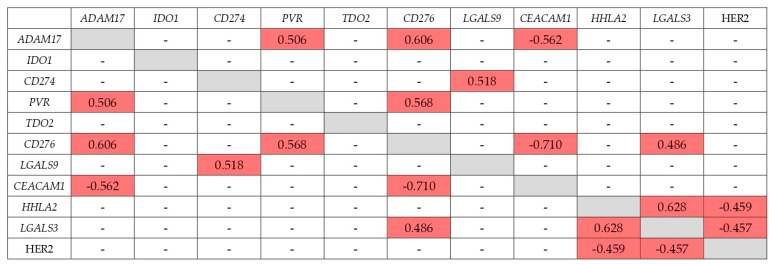
Correlation of expression at stage IV (metastatic GC). 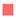 significant correlation; 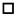 there is no significant correlation.

**Figure 4:**
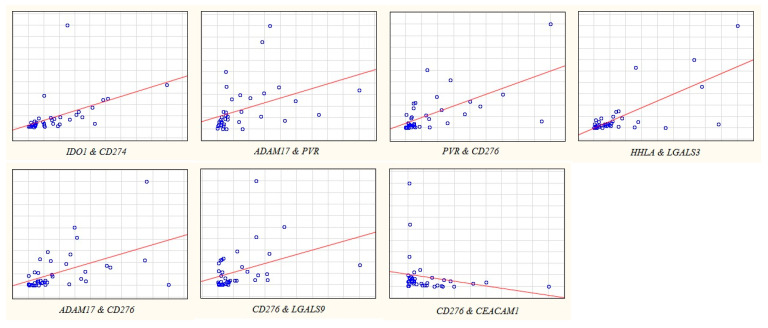
Scatterplot of gene expression at stage I + II (early GC).

**Figure 5:**
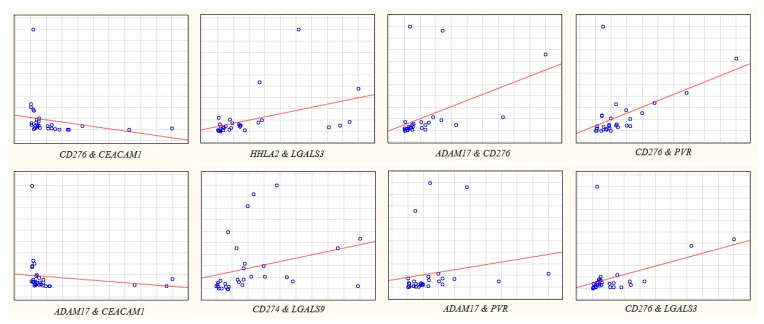
Scatterplot of gene expression at stage IV (metastatic GC).

In addition to the level of IC expression, an important feature that can determine the therapeutic efficacy of IC inhibition may be the expression of another IC that correlates with it. These features have still been poorly studied. To elucidate them, we revealed the expression correlation coefficients for all genes studied in this work and HER2, significant for GC therapy, at different stages of GC (Figure 2, Figure 3, Figure 4 and Figure 5).

Spearman’s correlation analysis (*p* < 0.01) showed that correlation of expression levels at stage IV looks similar to stage I + II (Figure 2, Figure 3, Figure 4 and Figure 5). The expression of the *CD276* gene showed the highest number of correlations: in the early stages with the *ADAM17*, *PVR*, *CEACAM1*, and *LGALS9* genes, R = −0.458–0.586; at stage IV with the *ADAM17*, *PVR*, *CEACAM1*, and *LGALS3* genes, R = 0.486–−0.710). *CD274* gene expression at stage I + II correlated only with *IDO1* expression (R = 0.653, the highest correlation coefficient at stage I + II), but at stage IV only with *LGALS9* expression (R = 0.518). Almost all of the genes studied, except *TDO2*, have at some stage other IC genes with co-expression. At stage IV, the expression of the *IDO1* gene does not correlate with any other gene. As a rule, the correlation is positive, although there are exceptions. The expression of the *CD276* and *CEACAM1* genes negatively correlates both at early and late stages. At stage IV *CEACAM1* is expressed ‘in antiphase’ with the *ADAM17* gene. The expression of the *CEACAM1* gene has only negative correlation coefficients with the expression of other genes. HER2 expression correlated with *HHLA2* and *LGALS3* genes at stage IV (R = −0.459 and −0.457, respectively), but none in the stage I + II.

### 2.3. The Relationship of IC Gene Expression with the Degree of Tumor Differentiation

Distant metastasis in GC is associated with poor prognosis. Other clinical and pathological characteristics, including tumor differentiation degree, also affect the prognosis [5]. In this regard, the relationship between IC gene expression and tumor differentiation degree was studied.

**Figure 6:**
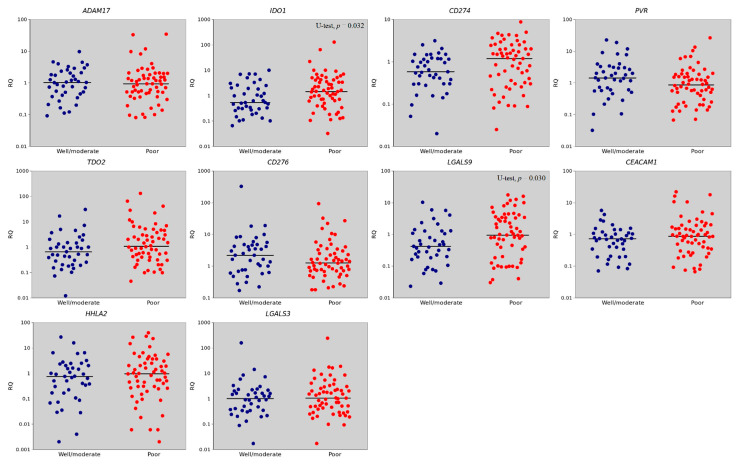
The relative gene expression (RQ) in poorly differentiated tumors (red dots) and well/moderately differentiated tumors (blue dots). Gene expression values are presented on a logarithmic scale. The line marks the median.

Figure 6 shows the values of the gene expression levels and the significantly different medians for *IDO1* and *LGALS9* genes in GC with well/moderate and poor degree of tumor differentiation.

ROC analysis revealed that the expression of the *IDO1* (*p* = 0.025) and *LGALS9* (*p* = 0.024) genes had a statistically significant relationship with the degree of tumor differentiation (Table 4).

Thus, in poorly differentiated tumors increased expression of the *IDO1* and *LGALS9* genes (relatively to well/moderately differentiated tumors) was observed. As it is known, the degree of tumor differentiation correlates with the type by Lauren classification: in diffuse type, a low differentiation degree is observed the most frequently. The Lauren type is also considered as one of the most important characteristics for GC prognosis [6,7]. Thereby, the relationship between the expression of the *IDO1* and *LGALS9* genes and the Lauren type was studied.

### 2.4. The Relationship of IC Gene Expression with the Lauren Type

To characterize the relationship between the expression levels of these genes and the Lauren type, ROC analysis was used. The analysis revealed that the expression of the *LGALS9* gene had a statistically significant relationship with the Lauren type (Table 5) with increased expression level in the diffuse/mixed type, while the expression of the *IDO1* gene was not associated with the Lauren type. In this regard, all analyzed IC genes were studied. ROC analysis revealed a significant *p*-value for the *CD274* gene (AUC = 0.645; sensitivity = 54.29; specificity = 85.29; *p*-value = 0.039), remaining genes did not show significant relationship with Lauren type (AUC ranged from 0.522 to 0.627; *p*-value ranged from 0.059 to 0.755).

We found a relationship between *LGALS9* gene expression both with the degree of tumor differentiation and Lauren type. Since these pathological characteristics are interconnected, in order to reveal which characteristic is basic, multiple logistic regression analysis was carried out (Table 6).

According to the results of the multiple regression analysis, the Lauren type is an independent feature. Thus, the expression of the *LGALS9* gene is associated with the Lauren type, while the degree of tumor differentiation is a secondary (tumor-type dependent) characteristic.

## 3. Discussion

A very significant factor influencing the therapeutic effect of IC inhibition may be the co-expression of another IC with respect to the inhibited one. Such co-expression may also be able to block the antitumor effect of the immune system, which would lead to the lack of a therapeutic effect of immunotherapy based on the inhibition of IC. In this work, for the first time, we studied the co-expression of a set of IC genes—*ADAM17*, *PVR*, *TDO2*, *CD274*, *CD276*, *CEACAM1*, *IDO1*, *LGALS3*, *LGALS9*, and *HHLA2*—in GC tumors at the early stages of its development and during metastasis.

As was found, 9 out of 10 genes studied have at some stage other IC genes with expression coordinated with them. The exception is the *TDO2* gene, whose expression does not correlate with the expression of other genes studied at all stages of GC. However, Cui et al. found a correlation between *TDO2* expression level and a number of IC genes and, including the *CD274*, *CD276*, and *IDO1* genes in a sample of GC from the TCGA database, that may be due to the difference in samples [8]. In metastatic GC, the expression of the *IDO1* gene does not correlate with any other gene. Except these genes, for the remaining eight there is a significant probability of co-expression of other IC genes. This is especially important to keep in mind for the *CD274* gene, encoding PD-L1, which is co-expressed with *IDO1* at early stage of GC and with *LGALS9* at late stage. Although the inhibition of these ICs has not yet been used in practice, they are being actively studied and have the prospect of entering the arsenal of immunotherapy tools [9,10].

Basically, the correlation of the expression of the genes studied is positive. An exception is the negative correlation between *CD276* and *CEACAM1* gene expression both at the early and metastatic stages. So far, the therapeutic efficacy of CEACAM1 inhibition has not been determined, but the sum of available results does not exclude such a prospect [1]. In this case, inhibition of CEACAM1 may be particularly beneficial in the absence of expression of B7-H3, encoded by the *CD276* gene. It should be noted that this gene has the highest number of expression correlations with other ICs among the ones studied. This feature may reduce the therapeutic efficacy of B7-H3 inhibition.

An essential characteristic of IC is the association of expression with tumor metastasis, which may be due to the blocking of the action of the immune system on cells detached from the tumor. On the other hand, differences in expression at different stages of tumor development may serve as an indication of the stage at which inhibition of this IC may be the most effective therapeutically. In our study, the expression levels of two genes were associated with GC metastasis—*TDO2* and *LGALS3*. TDO2 has one of the key roles in the catabolism of the Tryptophan to Kynurenine, along with IDO1 and IDO2. It is known that IDO1 catabolizes most of the tryptophan in various organs, while TDO2 is mainly expressed in the liver [11]. Increased TDO2 expression accelerates this process, leading to a decrease in the concentration of Tryptophan and an increase in the concentration of Kynurenine. This reduces the proliferation and activity of CD8+ T-cells and the strengthening of their apoptosis, contributing to the evasion of the tumor from the immune response [12]. This mechanism corresponds to our experimental results, in which increased TDO2 mRNA expression was first associated with distant metastasis in GC. In metastatic tumors TDO2 was expressed four times higher than in non-metastatic. In Pham et al. study, TDO2 expression was correlated with progression and outcome in GC [13]. Meta-analysis on prognosis and clinical features of TDO2 expression in various malignancies revealed that TDO2 overexpression has been associated with poor survival, TNM stage, and regional lymph node metastasis [14]. Moreover, bioinformatic analysis showed a correlation of high TDO2 expression with a poor prognosis in many cancer types [8]. The revealed features of TDO2—a significant increase in expression at the metastatic stage, the absence of other IC genes with correlated expression—indicate the prospect of inhibiting this gene in a metastatic GC.

Galectin-3 (Gal-3) is a member of the galectin family that is widespread in mammalian tissues and is determined by its carbohydrate recognition domains with a specific binding affinity for β-galactosides [15]. Gal-3, encoded by the *LGALS3* gene, is involved in the regulation of tumor cell growth, transformation, apoptosis, immunosuppression, angiogenesis, adhesion, invasion, and metastasis [16]. A decrease in Gal-3 expression reduces adhesion between tumor cells and facilitates the invasion of cancer cells [17]. Earlier, in the meta-analysis of Gal-3 expression in GC, measured by immunohistochemistry (IHC) method, the association of reduced Gal-3 expression with poor prognosis and high TNM stage was shown [18]. In our work, for the first time we have shown the association of reduced *LGALS3* gene expression in the tumor with a distant metastasis in GC. In metastatic tumors, *LGALS3* was expressed two-fold lower than in non-metastatic one. This suggests a greater therapeutic efficacy of inhibition of *LGALS3* in the early stages of GC in relation to metastatic tumors, if any.

IDO1 and TDO2 are intracellular metalloproteins that catalyze the first step of the kynurenine pathway that converts the essential amino acid tryptophan to kynurenine. IDO1 expression may be induced by IFN-γ. Overactivation of the kynurenine pathway results in a decrease in tryptophan and an increase in the kynurenine level. Accumulation of kynurenine is toxic to immune cells and can lead to arrest of cell cycle in CD8+ T-cells, NK-cells, and NKT-cells through the GCN and mTOR signaling pathways [19]. High expression of IDO1 in patients with GC is positively associated with tumor invasion and metastasis. In addition, increased IDO1 expression is associated with fewer number of CD4+ and CD8+ T-cells and higher number of Treg cells in tumors [20,21]. In GC, increased expression of IDO1 is associated with poor OS [20,22,23]. We found the association of increased expression of the *IDO1* gene with a poor differentiation in GC for the first time. The IDO1 may be considered for inhibition in tumors with a low differentiation degree, as connected with GC differentiation.

*CD274* gene encodes the PD-L1 IC, which is essential in the immune response. Interaction of PD-L1 with PD-1 receptor leads to inhibition of T-cell activation, CD8+ cytotoxic T-cells apoptosis, and increase of Foxp3+ Tregs number, which contributes the tumor to evade the immunity [24]. PD-L1 expression is regulated by several signaling pathways such as PI3K/AKT, MAPK, JAK-STAT, WNT, NF-κB, and Hedgehog [25]. PD-L1 is expressed in tumor cells of 30% of GC cases, but not in non-neoplastic gastric epithelium [26]. The expression of PD-L1 is being studied as a marker of poor prognosis in various types of malignancies [27,28,29]. Increased PD-L1 expression is also associated with a poor prognosis in GC [30,31,32]. We found an association of increased CD274 mRNA expression with a diffuse/mixed type according to the Lauren classification. Chen et al. study also showed that the expression of PD-L1, measured by the IHC method, correlated with the Lauren type [33]. *LGALS9* gene encodes Galectin-9 (Gal-9), another member of the galectin family. Gal-9 triggers the signaling pathways required for stimulation of innate immunity, recruits eosinophils and neutrophils to the site of infection, and facilitates the maturation of dendritic cells. Interaction of Gal-9 with receptors on the cell surface leads to the production of pro-inflammatory cytokines and chemokines by activated T-cells. Changes in intra- and extracellular Gal-9 concentration lead to physiological changes [34]. One of the Gal-9 receptors is Tim-3, an exhaustion marker that is expressed by activated T-cells. Binding of Gal-9 to Tim-3 leads to apoptosis of peripheral T-cells through the calcium-calpain-caspase-1 pathway. Most TIM-3+ T-cells in tumors co-express PD-1, another receptor of Gal-9. Gal-9–PD-1 binding contributes to the persistence of PD-1 + TIM-3+ T cells and attenuates Gal-9/Tim-3-induced cell death [9]. Gal-9 expression is significantly altered in most malignant tumors [35]. In solid tumors, increased expression of Gal-9 was significantly correlated with a lower depth of invasion, an earlier histopathological stage, the absence of lymph node metastases and the absence of distant metastases [36]. In GC some studies find better survival with increased expression of Gal-9, and there are also conflicting results in different studies [35,37,38]. 

In our study, elevated *LGALS9* expression was associated with poor differentiation and diffuse/mixed Lauren type. Furthermore, according to the results of multiple logistic regression, the Lauren type is an independent feature, while tumor differentiation degree is associated with the expression of *LGALS9* as a secondary characteristic. The feature found should be taken into account in further studies of this gene as a therapeutic target for immunotherapy. The expression of the IC genes studied does not correlate positively with the expression of HER2. This result indicates in favor of the possibility of independent use of inhibitors of the studied ICs and HER2.

## 4. Materials and Methods

### 4.1. Clinical Samples

The samples were obtained at the N.N. Blokhin National Medical Research Center of Oncology (N.N. Blokhin NMRCO). All samples underwent histological examination (including HER2 status) in the Department of Pathological Anatomy of Human Tumors at N.N. Blokhin NMRCO, and were clinically characterized. A total of 101 paired samples of gastric tissue (tumor tissue and morphologically normal tissue from the same stomach) were examined (Table 7). The samples were freshly frozen surgical or esophagogastroduodenoscopy material. The normal stomach tissue was used as a control. There were 54 males and 47 females with average age of 62 years (ranging from 27 to 85 years). The samples were classified using the 8th edition of the AJCC TNM classification from clinical stages I–IV. There were 10 (9.9%) stage I, 31 (30.7%) stage II, 29 (28.7%) stage III, and 31 (30.7%) stage IV samples. Because of the small number of stage I samples and the similarity of their clinical characteristics to stage II samples, the two sets of data were combined: stage I + II = stage I/II = 41 (40.6%).

### 4.2. Gene Expression Analysis

Expression of the following 10 IC genes were studied in 101 GC tumor samples: *ADAM17*, *PVR*, *TDO2*, *CD274*, *CD276*, *CEACAM1*, *IDO1*, *LGALS3*, *LGALS9* and *HHLA2*. Genes from each GC tumor sample were paired with and compared to those of a sample of normal tissue from the same stomach. 

The commercial RNeasy Mini Kit (Qiagen, Frederick, MD, USA) was used to isolate total RNA from tumor and normal stomach tissue samples. The presence and intensity of 28S/18S rRNA bands of the total RNA were checked via electrophoresis on a 1.8% agarose gel using the Bio-Rad Subcell horizontal electrophoresis chamber (Bio-Rad, Hercules, CA, USA) and gel imaging with the GelDoc XR+ imaging and gel documentation system (Bio-Rad, CA, USA). The quantity and quality of RNA was evaluated using the Nanodrop-ND 1000 UV–Vis Spectrophotometer (Thermo Fisher, Waltham, MA, USA). RNA was considered of acceptable quality if the two bands corresponding to 28S and 18S rRNA had an intensity ratio of ~2:1, and the A260/A280 ratio was 1.8–2.1. MMLV RT kit (Evrogen, Moscow, Russia) was used for the reverse transcription reaction. RT-PCR was performed using the qPCRmix-HS SYBR + HighROX Master Mix (Evrogen, Moscow, Russia). Quantitative RT-PCR (qRT-PCR) proceeded using the QuantStudio 5 Real-Time PCR System (Applied Biosystems, Foster City, CA, USA). RT-PCR was performed in triplicate for each gene along with a no-template negative control. The samples were amplified using a predenaturation phase of 5 min at 95 °C, followed by 45 cycles of 20 s at 95 °C, 20 s of primer annealing at 60 °C, and 20 s of extension at 72 °C. After the 45 cycles were completed, a melting curve analysis was performed. The *GAPDH* gene was used as an endogenous control. The relative level of mRNA expression of each gene was calculated in tumor tissues relative to normal stomach tissue using QuantStudio Design and Analysis Software (Applied Biosystems, CA, USA) for the ΔΔCt (RQ) method. The primer sequences for each gene and their predicted size are listed in Table 8.

### 4.3. Statistical Analysis

Statistical data processing was performed using Statistica 10.0 software, MedCalc program and the online calculator: https://www.medcalc.org/calc (accessed on 30 September 2022). Differences in the expression levels were evaluated using the U criterion; ROC analysis, Fisher’s exact test, and the logistic regression method were used to evaluate the relationship between the expression levels and clinicopathological characteristics. The significance level was established at *p* < 0.05.

Since we conducted a study on the association between clinicopathological characteristics and simultaneous expression of several genes, we applied the correction for the multiplicity of comparisons using the false discovery rate method (FDR). The application of this amendment avoids false ‘discoveries’ that might arise for statistical reasons in multiple comparisons. The significance level was set at *p* < 0.05.

## 5. Conclusions

For the first time, data were obtained on the correlation of the expression of 10 IC genes in GC at early stages of development and during metastasis. Almost all genes studied, except *TDO2*, have at some stage other IC genes with expression coordinated with them. This should be taken into account in the study and in the possible use of IC inhibition. Of particular interest in this regard is the co-expression of the *CD274* gene, which encodes PD-L1, at early stage of GC with *IDO1* and with *LGALS9* at late stage. It is not excluded, that correlation of PD-L1 expression with another ICs may be a cause of resistance to PD-L1 inhibition. At stage IV, the expression of the *IDO1* gene does not correlate with any other gene. This suggests that IDO1 inhibition at stage IV may be the most effective. As a rule, the correlation is positive, although there are exceptions. The expression of the *CD276* and *CEACAM1* genes negatively correlates both at the early and metastatic stages. The inhibition of CEACAM1 may be used in absence of B7-H3 expression, encoded by the *CD276*—gene with the highest number of expression correlations with other ICs. These data and their possible practical consequence accented the importance of parallel investigation of IC expression association with cancer development and IC expression coordination. 

In particular, the expression of the *TDO2* and *LGALS3* genes had a statistically significant relationship with GC metastasis. In metastatic tumors *TDO2* was expressed four-fold higher than in non-metastatic tumors, but *LGALS3* was two-fold lower. The differentiation was associated with another IC gene, *IDO1*. The expression of *CD274* (PD-L1) and *LGALS9* was associated with the type of GC by Lauren classification that should be considered in future studies and practical PD-L1 inhibition.

The revealed features of *TDO2*—a significant increase in expression at the metastatic stage, the absence of other IC genes with correlated expression—indicate the prospect of inhibiting this gene in a metastatic GC tumor. The IDO1, as connected with GC differentiation, may be considered for inhibition in tumors with a low differentiation degree.

## Figures and Tables

**Table 1:** The medians of expression and significance of their differences in GC samples with and without metastases.

Gene	Gene Name	The Median Value in the Non-Metastatic Group	The Median Value in the Metastatic Group	*p* = (Mann-Whitey U-Test)
*ADAM17*	A Disintegrin and Metalloproteinase Domain 17	0.91	1.19	0.198
*IDO1*	Indoleamine 2,3-Dioxygenase	0.99	1.10	0.726
*CD274*	Cluster of Differentiation 274	0.82	0.95	0.444
*PVR*	Poliovirus Receptor	1.23	1.19	0.657
*TDO2*	Tryptophan 2,3-Dioxygenase	0.76	3.03	0.024
*CD276*	Cluster of Differentiation 276	1.58	1.43	0.732
*LGALS9*	Galectin-9	0.66	0.93	0.202
*CEACAM1*	Carcinoembryonic Antigen-Related Cell Adhesion Molecule 1	0.76	0.91	0.405
*HHLA2*	Human Endogenous Retrovirus-H Long Terminal Repeat-Associating Protein 2	0.92	0.34	0.109
*LGALS3*	Galectin-3	1.51	0.85	0.031

**Table 2:** The relationship of gene expression with the development of metastases using ROC analysis.

Gene	Area under ROC Curve (AUC)/95% CI	Cut-Off Value	Sensitivity	Specificity	Significance Level *p* (Area = 0.5)	Benjamini-Hochberg Adjusted *p*-Value
*ADAM17*	0.584/0.462–0.706	>0.8	-	-	0.179	-
*IDO1*	0.525/0.392–0.657	>0.1	-	-	0.716	-
*CD274*	0.552/0.423–0.682	>0.5	-	-	0.428	-
*PVR*	0.529/0.407–0.651	≤3	-	-	0.639	-
*TDO2*	0.662/0.524–0.800	>1.6	68	76.09	0.021	0.042
*CD276*	0.522/0.398–0.645	≤0.3	-	-	0.729	-
*LGALS9*	0.582/0.467–0.698	>0.3	-	-	0.161	-
*CEACAM1*	0.553/0.428–0.678	>0.8	-	-	0.41	-
*HHLA2*	0.611/0.476–0.746	≤0.3	-	-	0.106	-
*LGALS3*	0.639/0.519–0.758	≤1.6	82.76	46.38	0.023	0.023

**Table 3:** The frequencies of gene expression level relative to the cut-off value in groups of patients with GC metastases and without metastases and the association of gene expression with metastasis by Fisher’s exact test.

Gene	Frequency of Expression Higher/Lower From the Cut-Off Value in Non-Metastatic GC	Frequency of Expression Higher/Lower From the Cut-off Value in Metastatic GC	Odds Ratio/95% CI	Relative Risk/95% CI	Fisher’s Exact Test, *p* =	Benjamini-Hochberg Adjusted *p*-Value
*ADAM17*	37/33	22/9	2.18/0.88–5.40	1.74/0.89–3.39	0.125	-
*IDO1*	62/8	31/0	8.57/0.48–153.27	6.03/0.40–90.54	0.102	-
*CD274*	42/28	24/7	2.29/0.87–6.02	1.82/0.87–3.79	0.114	-
*PVR*	19/51	3/28	3.48/0.95–12.78	2.60/0.87–7.75	0.067	-
*TDO2*	17/53	21/10	6.55/2.58–16.60	3.48/1.84–6.58	<0.001	<0.001
*CD276*	66/4	26/5	3.17/0.79–12.75	1.97/1.01–3.84	0.128	-
*LGALS9*	43/27	27/4	4.24/1.34–13.45	2.99/1.14–7.82	0.01	0.01
*CEACAM1*	33/37	19/12	1.78/0.75–4.20	1.49/0.81–2.74	0.204	-
*HHLA2*	48/22	15/16	2.33/0.98–5.54	1.77/0.99–3.15	0.075	-
*LGALS3*	32/38	5/26	4.38/1.51–12.72	3.01/1.26–7.16	0.007	0.011

**Table 4:** The relationship of gene expression with the degree of tumor differentiation—ROC analysis.

Gene	Area under ROC Curve (AUC)/95% CI	Cut-Off Value	Sensitivity	Specificity	Significance Level *p* (Area = 0.5)	Benjamini-Hochberg Adjusted *p*-Value
*ADAM17*	0.531/0.403–0.659	≤1.5	-	-	0.637	-
*IDO1*	0.644/0.518–0.769	>0.6	74.47	58.06	0.025	0.025
*CD274*	0.601/0.474–0.728	>1.2	-	-	0.118	-
*PVR*	0.622/0.498–0.745	≤1.0	-	-	0.054	-
*TDO2*	0.604/0.466–0.742	>0.5	-	-	0.141	-
*CD276*	0.566/0.440–0.692	≤1.8	-	-	0.303	-
*LGALS9*	0.635/0.518–0.752	>0.6	66.04	67.57	0.024	0.048
*CEACAM1*	0.570/0.452–0.688	>0.8	-	-	0.243	-
*HHLA2*	0.546/0.419–0.674	>3.2	-	-	0.476	-
*LGALS3*	0.539/0.419–0.659	>2.4	-	-	0.523	-

**Table 5:** The relationship of gene expression with the type by Lauren classification—ROC analysis.

Gene	Area under ROC Curve (AUC)/95% CI	Cut-Off Value	Sensitivity	Specificity	Significance Level *p* (Area = 0.5)	Benjamini-Hochberg Adjusted *p*-Value
*IDO1*	0.552/0.405–0.698	>0.6	68.42	48.28	0.489	0.489
*LGALS9*	0.766/0.659–0.873	>0.6	75.61	75.00	<0.0001	<0.0001

**Table 6:** Identification of independent feature for *LGALS9* gene expression—multiple logistic regression.

Characteristic	Coefficient	Standard Error	*p*-Value	Odds Ratio/95% CI
Differentiation	−0.326	0.851	0.702	0.72/0.14–3.84
Lauren type	2.475	0.843	0.003	11.88/2.28–62.00

**Table 7:** Patient characteristics.

Clinicopathological Features		Number of Patients
Gender	Male	54
	Female	47
Age	<60	34
	>60	67
Tumor location	Upper	19
	Middle	44
	Lower	35
	Whole	3
Differentiation	Well/moderate	41
	Poor	60
Lauren type	Intestinal	46
	Diffuse	45
	Mixed	9
	Non-classified	1
Depth of tumor invasion (T)	1	7
	2	10
	3	23
	4	61
Lymph node metastasis (N)	0	43
	1	22
	2	22
	3	14
Distant metastasis (M)	M0	70
	M1	31
Stage (8th AJCC)	IA	6
	IB	4
	IIA	9
	IIB	22
	IIIA	11
	IIIB	14
	IIIC	4
	IV	31

**Table 8:** Primer sequences for qRT-PCR and their amplicon size.

Gene	Primer Direction	Primer Sequence (From 5′→3′)	Product Size (bp)
*GAPDH*	Forward	GGCTGCTTTTAACTCTGG	190
	Reverse	GGAGGGATCTCGCTCC	
*ADAM17*	Forward	GCTTGGATCTTGGCAAGTGT	150
	Reverse	CATCGACATAGGGCACACAG	
*IDO1*	Forward	CCAGCTATCAGACGGTCTG	228
	Reverse	CGGACTGAGGGATTTGACTC	
*CD274*	Forward	GTGCCGACTACAAGCGAATT	104
	Reverse	TGTCAGTTCATGTTCAGAGGTG	
*PVR*	Forward	CTACACCTGCCTGTTCGTCA	186
	Reverse	TCTGAGTGCCAGGTGATTTG	
*TDO2*	Forward	TCCTCAGGCTATCACTACCTGC	110
	Reverse	ATCTTCGGTATCCAGTGTCGG	
*CD276*	Forward	GGCTGTCTGTCTGTCTCATTG	176
	Reverse	TCCATCATCTTCTTTGCTGTCA	
*LGALS9*	Forward	GATGAGAATGCTGTGGTCCG	260
	Reverse	GAAGCCGCCTATGTCTGCA	
*CEACAM1*	Forward	TCTACCCTGAACTTTGAAGCCCA	150
	Reverse	TGAGAGACTTGAAATACATCAGCACTG	
*HHLA2*	Forward	AGTGGTGCTAAAGGTGGGAGTT	154
	Reverse	CATGTTGTTTTCAGAGATAGGTGTGT	
*LGALS3*	Forward	GGCCACTGATTGTGCCTTAT	154
	Reverse	AAGCGTGGGTTAAAGTGGAAG	

## Data Availability

Not applicable.
